# Medium chain fatty acid supplementation improves animal metabolic and immune status during the transition period: A study on dairy cattle

**DOI:** 10.3389/fimmu.2023.1018867

**Published:** 2023-01-27

**Authors:** Zhonghan Wang, Qianqian Wang, Chuanlan Tang, Jing Yuan, Chenglong Luo, Dong Li, Tian Xie, Xiaoge Sun, Yan Zhang, Zhantao Yang, Cheng Guo, Zhijun Cao, Shengli Li, Wei Wang

**Affiliations:** ^1^ State Key Laboratory of Animal Nutrition, College of Animal Science and Technology, China Agricultural University, Beijing, China; ^2^ Animal Production Systems Group, Wageningen University & Research, Wageningen, Netherlands; ^3^ School of Agriculture, Ningxia University, Yinchuan, China

**Keywords:** transition, dairy cows, medium chain fatty acids, immunity, metabolism

## Abstract

The transition period is the stage of the high incidence of metabolic and infectious diseases in dairy cows. Improving transition dairy cows’ health is crucial for the industry. This study aimed to determine the effects of dietary supplementation medium-chain fatty acids (MCFAs) on immune function, metabolic status, performance of transition dairy cows. Twenty multiparous Holstein cows randomly assigned to two treatments at 35 d before calving. 1) CON (fed the basal 2) MCFA treatment (basal diet was supplemented at an additional 20 g MCFAs mixture every day) until 70 d after calving. The results showed that the serum amyloid A myeloperoxidase concentrations in the blood of cows in MCFA treatment significantly decreased during the early lactation (from 1 d to 28 d after calving) 0.03, 0.04, respectively) compared with the CON, while the tumor necrosis factor concentration was significantly decreased at 56 d after calving (*P* = 0.02). In addition, the concentration of insulin in the pre-calving (from 21 d before calving to calving) blood of cows in MCFA treatment was significantly decreased (*P* = 0.04), and concentration of triglyceride also showed a downward trend at 28 d after calving 0.07). Meanwhile, MCFAs supplementation significantly decreased the concentrations of lithocholic acid, hyodeoxycholic acid, and hyocholic acid in the blood at 1 d calving (*P* = 0.02, < 0.01, < 0.01, respectively), and the level of hyocholic acid taurocholic acid concentrations (*P* < 0.01, = 0.01, respectively) decreased dramatically at 14 d after calving. However, compared with the CON, the pre-calving dry matter intake and the early lactation milk yield in MCFA treatment were significantly decreased (*P* = 0.05, 0.02, respectively). In conclusion, MCFAs supplementation transition diet could improve the immune function and metabolic status of dairy cows, and the health of transition cows might be beneficial from the endocrine status.

## Introduction

The transition period is widely regarded as the most challenging period o the lactation cycle of dairy cows (1). To cope with various challenges, it is necessary to use the integrated adaptation mechanism coordinated by the endocrine system and the immune system (2, 3). However, adaptive ability varies among individual dairy cows (4), and different degrees of immunosuppression might occur, further resulting in infectious and metabolic diseases (5).

The lack of energy in transition (6, 7) results in the mobilization of body fat (8). Non-esterified fatty acid (NEFA) (9), triglyceride (TG), and ketone bodies are produced in this process (10), which affects liver metabolism, resulting in complex and difficult-to-regulate systemic responses (11). Meanwhile, the increase in the feed energy density of post-partum cows may lead to (12) a decrease in dry matter intake (DMI) and feed efficiency (13). Studies have shown that decreased DMI and increased negative energy balance (NEB) could trigger competition for essential nutrients between the immune system and milk generation. The lack of energy will impact immune response and immune cell function, resulting in immune suppression and increased disease susceptibility (14). Meanwhile, transition immunosuppression and excessive inflammatory responses are predisposing factors for metabolic disorders (15). Therefore, the disturbance of nutrient metabolism and immune homeostasis in dairy cows may cause harmful feedback loops, which affect the health and performance of dairy cows (2).

MCFAs (Medium chain fatty acids) are fatty acids containing 6-12 carbon atoms and are found mainly in coconut oil and palm kernel oil (16). After ingestion, MCFAs are absorbed by the gastrointestinal epithelial cells, some of which can be absorbed by the epithelial cells to enhance intestinal integrity (17), and some can enter the liver through the portal vein and directly metabolize. Besides, MCFAs can regulate the metabolism of carbohydrates and lipids in the liver (18) and promote the formation of liver glycogen (19) and the synthesis and excretion of hepatic bile acids (20, 21). In addition, MCFAs can regulate the release of inflammatory cytokines, such as IL-6 and IL-8, preventing inflammation and decreasing the damage caused by inflammation (22).

Currently, the application of MCFAs in animal production is mainly carried out on monogastric animals. It was reported that MCFA supplementation could improve DMI, feed efficiency, and the average daily gain of nursery pigs (23). The application values of MCFAs in the monogastric animal are attributed to their antipathogen activity and immune-improving reaction (24, 25). However, the application values of MCFAs in ruminants are still controversial. Relevant studies showed that MCFA supplementation could improve fat digestibility (26) and promote milk fat synthesis in lactating dairy cattle (27, 28). Nevertheless, other studies on dairy cows and swamp buffalo have reported that MCFAs decrease the digestibility of fiber, which leads to a decline in DMI (29, 30). Besides, several cell studies have shown that MCFAs have antipathogen (31) and anti-inflammatory activities (32). It carries out the relevant functions by stimulating cellular immunity and supporting transition cows to face various challenges (33), such as reducing the incidence rate of mastitis (34, 35). Moreover, there are few studies on MCFAs in transition dairy cows (36), so it is necessary for us to explore the application prospects of MCFAs in peripartum cows.

Overall, our hypothesis focuses on whether MCFAs could improve the immune function and metabolic status of dairy cows in the transition period and whether MCFA supplementation could modify dairy cows’ rumen fermentation and performance.

## Materials and methods

Animals involved in this experiment were fed according to the guidelines from the committee of animal welfare and animal experimental ethical inspection of China Agricultural University. The committee reviewed and approved the experiment and all procedures involving animals (Protocol number: CAU20201024-2).

### Cows, housing, feeding management,and treatment

Twenty healthy multiparity Holstein dairy cows with similar body condition scores (parity = 2.50 ± 1.42; body condition score = 3.28 ± 0.23; mean ± SD) were randomly assigned to two treatments, with 10 cows in each treatment. The control group (CON; parity = 2.67 ± 1.32; body condition score = 3.33 ± 0.28) was fed a basal diet, while the transition diet was followed in the pre-calving stage (21 d before calving to calving) and the lactation diet was followed in the early lactation (1 d to 28 d after calving) and peak lactation diet (29 d to 70 d after calving) (**Table 1**). The MCFA treatment (MCFA; parity = 2.33 ± 1.58; body condition score = 3.22 ± 0.15) was supplemented with 20 g of MCFA mixture consisting of 10% octanoic acid, 7% decanoic acid, 17% lauric acid, and 66% carrier (consisting of silica, wheat flour, and barley flour).

**Table 1:** Ingredient and chemical composition of the TMR.

Composition	The peripartum diet^1^	The lactating diet^2^
Ingredient (%DM)		
Oat grass	32.6	——
Alfalfa hay	——	7.1
Corn silage	46.6	56.6
Corn	4.2	7.1
Steam-flaked corn	——	9.9
Corn gluten meal	2.8	1.7
Bean pulp	4.6	8.5
Soy hulls	5.1	2.8
Whole cottonseed	——	1.2
Molasses	——	2.4
Diamond V XP^6^	0.2	0.1
Bergafat T 300^7^	——	0.8
Sodium bicarbonate	——	0.4
Urea	0.4	——
premix	3.2^3^	1.4^4^
Chemical composition (% DM)		
CP^5^	16.56	16.98
ADF^5^	27.62	14.73
NDF^5^	41.32	23.32
Lignin	3.81	2.45
Starch	13.27	29.61
Fat	1.91	3.77
peNDF^5^	33.38	15.61
Ca	1.45	0.86
P	0.27	0.38
K	1.04	1.31
DM^5^ (%)	50.19	54.4
${\text{NE}}_{L}^{5}$ (Mcal/kg)	1.44	1.78

NE_L_: a calculated value according to NRC (2001) (18), while the nutrient levels were measured values.

^1^Applied in the pre-calving cows.

^2^Applied in the early lactation and peak lactation cows.

^3^The transition diet premix contained 150000 - 350000 IU/kg Vitamin A, 30000 - 95000 IU/kg VitaminD_3_, ≥ 2500 mg/kg Vitamin E, 200 - 7500mg/kg Fe, 100 - 350 mg/kg Cu, 300 - 2500 mg/kg Mn, 400 - 2500 mg/kg Zn, 2 - 20 mg/kg Co.

^4^The lactating diet premix contained 70000 - 150000 IU/kg Vitamin A, 20000 - 50000 IU/kg Vitamin D, ≥ 400 mg/kg Vitamin E, 100 - 350 mg/kg Cu, 120 - 2000 mg/kg Mn, 400 - 2000 mg/kg Zn, 2 – 10 mg/kg Co.

^5^CP, crude protein; ADF, acid detergent fiber; NDF, neutral detergent fiber; pe NDF, physically effective neutral detergent fiber; DM, dry matter; NE, net energy.

^6^Diamond V XP (Diamond V, USA) contained proteins, peptides, antioxidants, organic acids, nucleotides, vitamins, minerals, beta-glucans, and mannan oligosaccharides.

^7^Bergafat T 300 (Berg-Schmidt, Germany) contained natural glycerin 10% and palmitic acid 75 - 93%.

The experiment was carried out in a barn equipped with automatic feeding bins (RIC, Roughage Intake Control, Insentec B.V. Marknesse). The barn was divided into two parts before the experiment (Barn 1 and Barn 2) to separate the transition and lactating cows. Cows were transferred to Barn 1 35 d before calving and 14 d for the adaptive phase and remained there until calving. After calving, they were transferred to Barn 2 until the end of the experiment. Both Barn 1 and Barn 2 were equipped with adequate feeding bins, cubicles, and water bins, providing cows with a comfortable environment, guaranteeing the experiment’s success and the animals’ welfare. No additional MCFA mixture was added to the cows’ diet during these two days as the cows needed to be taken care of in the calving room after calving, which took 1-2 d.

### Animal performance

The feed intake of dairy cows was recorded by the automatic RIC system. TMR samples were collected weekly before calving and every two weeks during the lactation period. After the feed samples were collected and weighed, they were dried in a forced-air-drying oven at 55 degrees for 48 hours (Senxin experimental instrument series DGG-9003, Shanghai, China). The dried samples were then equilibrated at ambient temperature and humidity before reweighing. Each cow wore a collar that recorded its rumination behavior after calving. An electronic monitoring system (HR-TAG-LD, SCR Engineers Ltd) recorded data every two hours to monitor the cow’s daily rumination time.

At the 6^th^, 12^th^, 18^th^, and 24^th^ hours of the last 3 d (68 d - 70 d after calving), 300 - 500 g feces were collected from the rectum, and the fecal samples were mixed with 10% tartaric acid and dried in a forced-air-drying oven at 55 degrees for 48 h (37). The dried TMR and feces samples were ground using a high-speed vertical grinder (RT-34, Kunjie Yucheng Machinery Equipment, Beijing, China) and then passed through a 1 mm mesh screen. Sieved TMR and feces samples were collected and tested for crude protein (CP) (Dumas combustion, Rapid MAX N Exceed, Elementar Analysensysteme GmbH, Hanau, Germany), ether extract (EE) (ANKOM XT15Ii, Extractor, ANKOM Technology, Macedon, America), neutral detergent fiber (NDF), and acid detergent fiber (ADF) (38) (ANKOM 2000Ii, Automated Fiber Analyzer, ANKOM Technology, Macedonia, USA), and the digestibility of each nutrient was calculated (39).

Daily milk production was recorded using an automatic milking system (ALPROTM, DeLaval, Sweden Tumba) for 70 d after calving. On days 14, 28, 42, 56, and 70 after calving, 50 mL milk was collected at the ratio of morning: afternoon: night (4:3:3). After mixing, 50mL milk was immediately sent to the lab to determine the content of milk fat, milk protein, and lactose, as well as the levels of urea nitrogen and the somatic cell count (SCC) (near-infrared reflectance spectroscopy, Series300 combi-foss; Foss Electric, Schiller ød, Denmark). Energy-corrected milk (ECM = 0.327× kg/d of milk+12.95× kg/d of milk fat yield+7.2× kg/d of milk protein yield) and fat-corrected milk (FCM = 0.4× kg/d of milk yield+15×milk fat %× kg/d of milk yield) were calculated according to milk composition. The feed efficiency of lactation was calculated by FCM/DMI.

### Rumen fluid collection and analysis

On days 21 and 7 before calving and 1 d, 14 d, 28 d, 42 d, 56 d, and 70 d after calving before morning feeding, 50 mL rumen fluid was extracted by oral intubation, filtered by four layers of gauze, and its pH value was determined immediately (SEVEN2Go portable pH meter, METTLER TOLEDO, Switzerland). The filtered rumen fluid was centrifuged at 5400 g/min for 10 min, 1 mL of supernatant was taken, and 0.2 mL 25% metaphosphoric acid solution containing standard internal 2 ethylbutanoic acid (2-EB) was added and mixed. Gas chromatography (6890N; Agilent Technologies, Avondale, PA, USA) and capillary column (HP-Innowax 19091N-213, Agilent) for determination of acetate, propionate, iso-butyrate, butyrate, iso-valerate and valerate acid in rumen fluid. Ammonia nitrogen (NH_3_-N) content was determined by the phenol-sodium hypochlorite colorimetric method (40)

### Blood sampling and analysis

Blood samples were taken from the tail vein of six randomly selected dairy cows 21 d and 7 d before calving and 1 d, 28 d, 56 d, and 70 d after calving. Before morning feeding, 10 mL of blood was collected from blood vessels containing anticoagulant EDTA. After centrifugation at 300 g for 15 min, upper plasma was collected and stored at -20 degrees until analysis. Insulin (INS) was determined by radioimmunoassay (BFM-96, multi-tube radioimmunocounter, Zhongcheng Electromechanical Technology). Plasma TG was determined by an automatic biochemical analyzer (CLS880, Jiangsu Zecheng Biotechnology). NEFA, β -hydroxybutyric acid (BHBA), immunoglobulin G, A, and M (IgG, IgA, IgM), interleukin (IL-2, IL-6, IL-8, IL-10), tumor necrosis factor (TNF-α), myeloperoxidase (MPO), and serum amyloid A (SAA) were detected using the ELISA method (fully automated ELISA machine, THERMO Multiskan Ascent) (41, 42).

The bile acids in the blood samples were extracted and purified by liquid chromatography-tandem mass spectrometry with internal isotope standard and then separated by BEHC18 ultra-high liquid chromatographic column. Gradient elution was performed with 0.1% formic acid-water and 0.1% formic acid-acetonitrile as mobile phases, and mass spectrometry was performed with negative ion mode. At the same time, a variety of isotope-labeled bile acids were used as an internal standard to correct errors in sample pretreatment and mass spectrometry analysis (43).

### Phagocytic ability of neutrophils

The tail vein blood of five dairy cows in the two treatments was collected using an EDTA tube 14 d, 28 d, 42 d, 56 d, and 70 d after calving. Neutrophils were isolated from the EDTA anticoagulated tube within 3 hours using the bovine peripheral blood neutrophil isolation kit (P9400, Solarbio, China). The isolated neutrophils were then added to 500 μL cell culture medium (RPMI-1640). After mixing well, viable cell concentrations were recorded with a cell counter (TC20TM, Bio-Red, America). Approximately 1×10^6^ neutrophils were absorbed according to the concentration, and the equivalent fluorescent microspheres (F8821, ThermoFisher, China) were added, mixed, and cultivated for 30 min at 37°C. Then, 200 μL of paraformaldehyde was added (blown and mixed) fixed for 30 min, and centrifuged at 1200 g for 8 min. The supernatant was discarded, and 350 μL of FACS (1 μL fetal bovine serum + 49 μL phosphate buffer saline) was added and transferred to a flow cytometry tube after passing through a 70-μm pore size cell filter, the flow cytometry was then used to analyze the samples (early lactation, BD FACS Calibur, BD Company, America) (peak lactation, BD FACS Verse, BD Company, America). Data were analyzed with FlowJo software and presented with histograms, using the fluorescent channel, PE as the abscissa. The parameter PE provided an accurate measurement of the brightness of the stained cells. Subsequent statistical analysis was performed according to the percentage of neutrophils that phagocytosed the fluorescent microspheres (44, 45).

### Statistical analysis

SAS Studio (SAS institute, Carry, NC, USA) software was used for statistical analysis. Before analysis, SAS was used to test the normal distribution of the data, and all the data were in line with normal distribution except SCC. The SCC results were then converted into SCC scores by log. Due to the great physiological changes of cows during the transition period, the experiment was divided into three stages for separate analysis: pre-calving (21 d before calving to calving), early lactation (1 d to 28 d after calving), and peak lactation (29 d to 70 d after calving). One-way ANOVA was used to analyze the nutrient digestibility of feed in the peak lactation stage of dairy cows. All other data were analyzed using the MIXED model of SAS, as follows:


$$Y_{ijk}=\mu +\alpha_{i}+\beta_{j}+{\left(\alpha \beta \right)}_{ij}+e_{ijk}$$

Where Y_ijk_ is the dependent variable, μ is the overall mean, αi is the treatment effect (i=1, 2), β_j_ is the effect of sampling time for the pre-calving cows, the early lactation cows, or the peak lactation cows, (αβ)_ij_ is the interaction effect of treatment and sampling time, and e_ijk_ is the residual error.

By status, “week” was treated as a repeated measure and “cow” as the subject of the repeated statement. Significance was declared at *P* ≤ 0.05, and tendencies were reported if 0.05< *P*≤ 0.10. If the P-value of an interaction term was ≤ 0.05, it was retained; otherwise, interaction terms were removed from the model.

## Results

### Animal performance

Dietary supplements of MCFAs significantly reduced the DMI in the pre-calving cows (*P* = 0.05). And in early lactation, milk yield in MCFA treatment was significantly lower than that in CON (*P* = 0.02), and ECM also had a decreasing trend (*P* = 0.06) (**Table 2**).

**Table 2:** Effects of feeding medium chain fatty acid on the performance of pre-calving, early lactation, and peak lactation cows^2^.

Variable	Treatment^1^		SEM	*P*-value		
	CON	MCFA		Treatment	Time	Treatment ×Time
Pre-calving^2^						
DMI^3^ (kg/d)	9.99^a^	8.77^b^	0.326	0.05	0.01	0.99
Early lactation^2^ (kg/d)						
DMI	15.02	14.18	0.608	0.42	<0.01	0.38
Milk yield	37.37^a^	32.56^b^	1.285	0.02	<0.01	0.38
4% FCM^3^	41.01	37.21	1.217	0.10	0.03	0.46
ECM^3^	43.62	38.78	1.351	0.06	0.04	0.37
Efficiency^3^	2.76	2.74	0.101	0.92	0.51	0.60
Peak lactation^2^ (kg/d)						
DMI	23.25	22.14	0.585	0.29	<0.01	0.78
Milk yield	47.01	43.44	0.977	0.09	0.93	0.99
4% FCM	47.59	47.11	1.119	0.82	0.04	0.60
ECM.	48.65	50.66	1.515	0.51	0.11	0.34
Efficiency	2.04	2.07	0.055	0.80	0.02	0.23

SEM, standard error of mean.

^a-b^ Means within a row with unlike superscripts differ (P ≤ 0.05).

^1^CON, control treatment, basic diet with no medium chain fatty acids, n = 9. MCFA = MCFA treatment, basic diet and medium chain fatty acids 20 g/d, n = 9.

^2^Pre-calving, from 21 d before calving to calving, early lactation = from 1 d to 28 d after calving, peak lactation = from 29 d to 70 d after calving.

^3^DMI, dry matter intake, FCM, fat corrected milk; ECM, energy corrected milk; Efficiency, FCM/DMI.

Milk composition analysis showed that milk fat has an increasing trend in early lactation (*P* = 0.09), while milk protein yield and lactose yield decreased significantly (*P<* 0.01, = 0.03, respectively) (**Table 3**). In peak lactation, milk lactose and milk fat percentage saw an interaction of time and treatment (*P* = 0.01, 0.07, respectively). MCFA supplementation significantly increased lactose percentage 42 d (*P* = 0.04) but significantly inhibited it 56 d after calving (*P* = 0.03). Milk fat percentage showed an increasing trend 56 d after calving (*P* = 0.09) (**Table 3**). The addition of MCFAs did not significantly influence the digestibility of each nutrient (**Table 4**).

**Table 3:** Effects of feeding medium-chain fatty acid on milk composition of early lactation and peak lactation cows^2^.

Variable	Treatment^1^		SEM	*P*-value		
	CON	MCFA		Treatment	Time	Treatment ×Time
Early lactation						
Milk fat percentage (%)	4.62	5.07	0.152	0.09	<0.01	0.49
Milk protein percentage (%)	3.28	3.11	0.063	0.13	<0.01	0.43
Mile lactose percentage (%)	5.17	5.18	0.034	0.94	0.37	0.76
Milk fat yield (kg/d)	1.71	1.62	0.053	0.42	0.27	0.72
Milk protein yield (kg/d)	1.24^a^	1.03^b^	0.044	<0.01	0.03	0.24
Milk lactose yield (kg/d)	1.98^a^	1.76^b^	0.071	0.03	<0.01	0.62
MUN^3^ (mg/dL)	19.76	17.63	1.014	0.32	0.36	0.88
SCC^3^ (×1000/mL)	197.07	43.00	41.458	0.32	0.09	0.72
Peak lactation						
Milk fat percentage (%)	4.36	4.61	0.142	0.31	0.01	0.07
Milk protein percentage (%)	3.24	3.22	0.038	0.85	0.26	0.59
Milk lactose percentage (%)	5.28	5.25	0.022	0.48	0.61	0.01
Milk fat yield (kg/d)	1.90	2.01	0.059	0.32	0.01	0.26
Milk protein yield (kg/d)	1.48	1.42	0.034	0.46	0.88	0.42
Milk lactose yield (kg/d)	2.46	2.28	0.056	0.11	0.56	0.13
MUN (mg/dL)	19.29	18.88	0.795	0.80	0.32	0.29
SCC (×1000/mL)	69.00	57.18	13.909	0.12	0.52	0.22

SEM, standard error of mean.

^a-b^Means within a row with unlike superscripts differ (P ≤ 0.05).

^1^CON, control treatment, basic diet with no medium chain fatty acids, n = 9. MCFA, MCFA treatment, basic diet and medium chain fatty acids 20 g/d, n = 9.

^2^Early lactation = from 1 d to 28 d after calving, Peak lactation, from 29 d to 70 d after calving.

^3^MUN, milk urea nitrogen, SCC, somatic cell count.

**Table 4:** Effects of feeding medium-chain fatty acid on nutrient digestibility of the peak lactation cows.

Digestibility (%)	Treatment		*SEM*	*P-value*
	CON	MCFA		
DM^3^	68.56	67.90	1.427	0.82
Protein	66.02	66.56	1.652	0.88
Fat	70.04	74.43	1.661	0.20
NDF^3^	27.74	29.09	3.403	0.87
ADF^3^	25.83	24.37	3.445	0.84

SEM, standard error of mean.

^a-b^Means within a row with unlike superscripts differ (P ≤ 0.05).

^1^CON, control treatment, basic diet with no medium chain fatty acids, n = 9. MCFA = MCFA treatment, basic diet and medium chain fatty acids 20 g/d, n = 9.

^2^Peak lactation, from 29 d to 70 d after calving.

^3^ADF, acid detergent fiber, NDF, neutral detergent fiber, DM, dry matter.

### Inflammatory marker, immune globulin, and phagocytic ability in blood

In the pre-calving period, there was a significant time × treatment interaction for γ-IFN (*P* = 0.04). However, there was no difference in γ-IFN at each time point before calving (**Table 5**). In early lactation, compared with CON, the concentrations of MPO and SAA in the serum of cows undergoing MCFA treatment were significantly decreased (all *P<* 0.05, **Table 5**). There was also an interaction trend of IL-6 (*P* = 0.06). Compared with the CON, the concentration of IL-6 in the blood of cows under MCFA treatment was significantly increased 1 d after calving (*P* = 0.04, **Table 5**). In peak lactation, TNF-α saw a significant interaction between time and treatment (*P* = 0.01) and decreased significantly 56 d after calving (*P* = 0.02) (**Table 5**).

**Table 5:** Effects of feeding medium chain fatty acid on blood neutrophils phagocytosis of early lactation cows and peak lactation cows^2^.

Variable	Treatment^1^		SEM	*P*-value		
	CON	MCFA		Treatment	Time	Treatment ×Time
Pre-calving						
IL^3^-2 (pg/mL)	152.05	152.33	2.202	0.57	0.04	0.29
IL-6 (ng/mL)	382.09	385.57	5.446	0.75	0.10	0.33
IL-8 (ng/mL)	82.65	80.34	1.074	0.28	0.07	0.51
IL-10 (pg/mL)	197.46	204.69	3.026	0.23	0.10	0.44
TNF-α^3^ (pg/L)	228.75	217.82	3.318	0.12	0.28	0.80
MPO^3^ (U/L)	125.48	126.08	1.253	0.83	0.78	0.82
SAA^3^ (μg/mL)	32.54	31.62	0.649	0.47	0.11	0.21
Early lactation						
IL-2 (pg/mL)	159.17	160.12	2.338	0.85	0.30	0.40
IL-6 (ng/mL)	405.38	415.11	7.003	0.42	<0.01	0.06
IL-8 (ng/mL)	88.02	91.39	1.519	0.29	0.31	0.29
IL-10 (pg/mL)	214.44	213.98	3.375	0.92	<0.01	0.21
TNF-α (pg/L)	234.38	234.14	2.655	0.97	0.33	0.78
MPO (U/L)	140.22^a^	133.81^b^	1.526	0.04	0.28	0.13
SAA (μg/mL)	32.24^a^	29.92^b^	0.561	0.03	0.01	0.29
Peak lactation						
IL-2 (pg/mL)	135.11	138.74	3.171	0.59	0.38	0.43
IL-6 (ng/mL)	416.84	436.53	9.149	0.32	0.56	0.69
IL-8 (ng/mL)	97.96	96.51	1.614	0.68	0.66	0.42
IL-10 (pg/mL)	231.24	230.32	3.373	0.89	0.06	0.82
TNF-α (pg/L)	238.10	229.13	4.321	0.25	0.27	0.01
MPO (U/L)	132.43	141.04	3.049	0.19	0.51	0.48
SAA (μg/mL)	31.06	30.58	0.820	0.76	0.05	0.14

SEM, standard error of mean.

^a-b^Means within a row with unlike superscripts differ (P ≤ 0.05).

^1^CON, control treatment, basic diet with no medium chain fatty acids, n = 9. MCFA = MCFA treatment, basic diet and medium chain fatty acids 20 g/d, n = 9.

^2^Pre-calving, from 21 d before calving to calving, Early lactation = from 1 d to 28 d after calving, Peak lactation= from 29 d to 70 d after calving.

^3^IL, interleukin, TNF- α, tumor necrosis factor α; MPO, myeloperoxidase; SAA, serum amyloid protein A.

There was an interaction trend for IgM (*P* = 0.09) in the pre-calving stage. IgM was significantly decreased 21 d before calving compared with the CON treatment (*P* = 0.02, **Table 6**). In early lactation, there was a significant interaction between time and treatment of IgM and IgA (*P*= 0.03,0.03, respectively). IgA concentration was also significantly decreased 28 d after calving (*P* = 0.01, **Table 6**), but IgM concentration was significantly increased 14 d after calving (*P* = 0.02, **Table 6**).

**Table 6:** Effects of feeding medium chain fatty acid on blood immune globulin of the pre-calving, early lactation, and peak lactation cows^2^.

Variable	Treatment^1^		SEM	*P*-value		
	CON	MCFA		Treatment	Time	Treatment×Time
Pre-calving						
Ig^3^-G (mg/mL)	7.53	7.56	0.170	0.96	0.15	0.21
Ig-A (μg/mL)	93.61	91.24	1.450	0.41	0.10	0.27
Ig-M (mg/mL)	0.83	0.78	0.013	0.03	0.03	0.09
Early lactation						
Ig-G (mg/mL)	8.12	8.30	0.209	0.66	0.01	0.70
Ig-A (μg/mL)	102.74	99.79	1.327	0.24	0.22	0.03
Ig-M (mg/mL)	0.80	0.82	0.017	0.53	0.02	0.03
Peak lactation						
Ig-G (mg/mL)	8.24	8.08	0.191	0.56	<0.01	0.26
Ig-A (μg/mL)	97.79	94.70	1.797	0.41	0.37	0.30
Ig-M (mg/mL)	0.86	0.82	0.017	0.34	0.71	0.73

SEM, standard error of mean.

^1^CON, control treatment, basic diet with no medium chain fatty acids, n = 9. MCFA, MCFA treatment, basic diet and medium chain fatty acids 20 g/d, n = 9.

^2^Pre-calving, from 21 d before calving to calving, Early lactation, from 1 d to 28 d after calving, Peak lactation,= from 29 d to 70 d after calving.

^3^Ig, Immune globulin.

**Figure 1:**
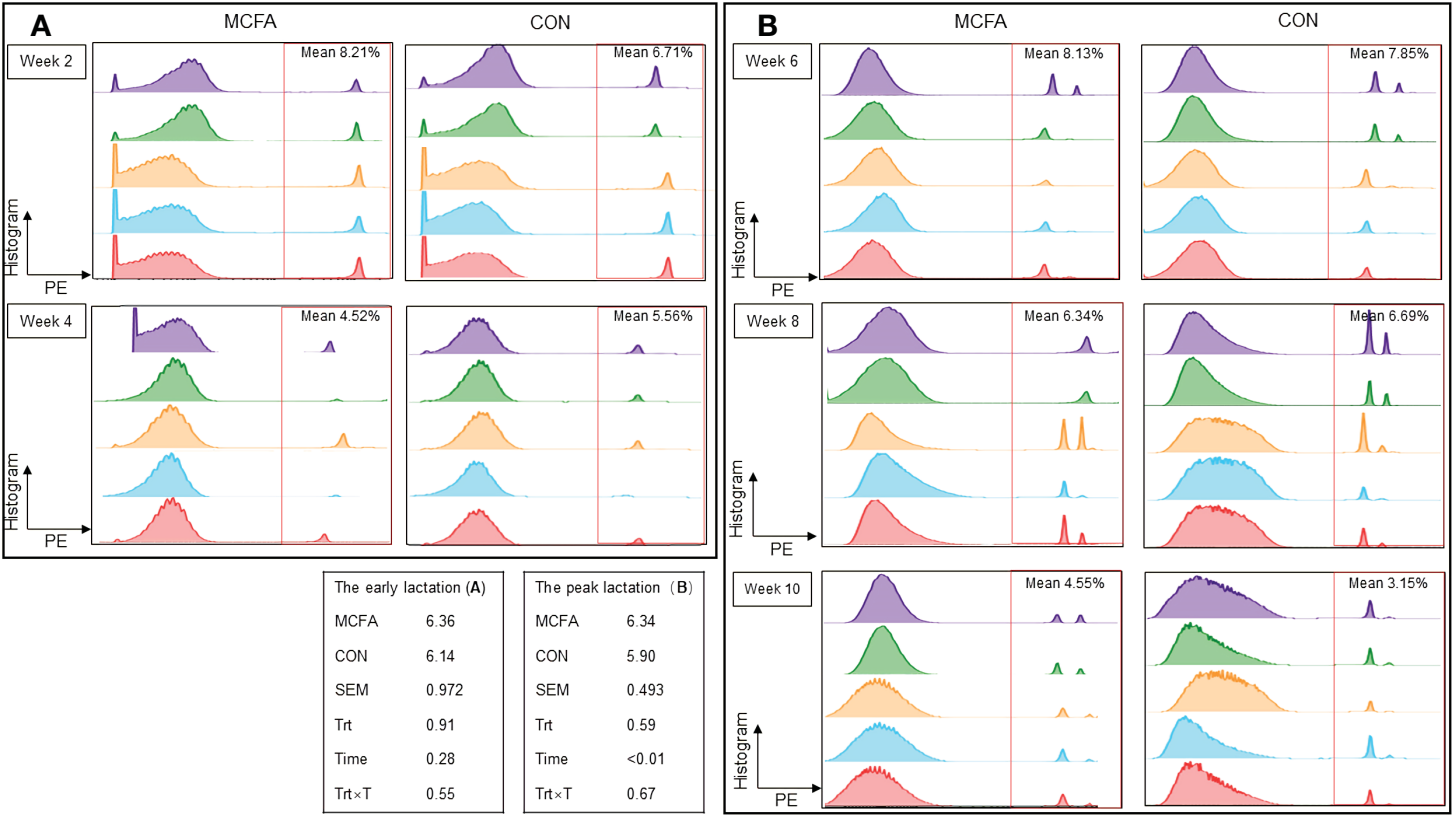
Effects of feeding medium chain fatty acid on blood neutrophils phagocytosis of the early lactation cows and the peak lactation cows. **(A)** The blood neutrophils phagocytosis of the early lactation dairy cows; **(B)** the blood neutrophils phagocytosis of the peak lactation dairy cows. CON, control treatment, n = 9; MCFA: MCFA treatment (basic diet and medium chain fatty acids 20 g/d), n = 9. SEM, standard error of mean. Trt, treatment effect; Time, the calving time effect; Trt × T, the interaction effect between treatment effect and time effect. The early lactation: from 1d to 28d after calving; the peak lactation: from 29 d to 70 d after calving.

Furthermore, there was no significant difference in neutrophils phagocytosis during early lactation and peak lactation (**Figures 1A, B**).

### Serum energy, insulin, and bile acid metabolism

The results of related metabolites and hormones in blood during the pre-calving period show that INS concentration was significantly reduced (*P* = 0.04; **Table 7**). TG showed a trend of interaction (*P* = 0.08) during early lactation, which had a decreasing trend compared with CON 28 d after calving (*P* = 0.07, **Table 7**).

**Table 7:** Effects of feeding medium-chain fatty acid on blood metabolites and hormones of pre-calving, early lactation, and peak lactation cows^2^.

Variable	Treatment^1^		SEM	*P*-value		
	CON	MCFA		Treatment	Time	Treatment×Time
Pre-calving						
BHBA^3^ (mmol/L)	0.48	0.47	0.007	0.62	0.05	0.43
NEFA^3^ (umol/L)	43.14	41.88	0.586	0.18	<0.01	0.20
TG^3^ (mmol/L)	0.23	0.20	0.001	0.10	0.05	0.84
INS^3^ (mIU/mL)	22.65^a^	18.47^b^	0.939	0.04	0.32	0.65
Early lactation						
BHBA (mmol/L)	0.50	0.50	0.007	0.62	0.01	0.11
NEFA (umol/L)	44.77	43.03	0.593	0.11	0.01	0.14
TG (mmol/L)	0.13	0.13	0.005	0.19	0.62	0.08
INS (mIU/mL)	14.13	14.98	0.681	0.54	0.10	0.41
Peak lactation						
BHBA (mmol/L)	0.46	0.44	0.009	0.34	0.97	0.92
NEFA (umol/L)	44.38	43.61	0.688	0.61	0.64	0.69
TG (mmol/L)	0.13	0.15	0.008	0.27	0.16	0.84
INS (mIU/mL)	17.01	18.19	1.028	0.42	<0.01	0.82

SEM, standard error of mean.

^a-b^Means within a row with unlike superscripts differ (P ≤ 0.05).

^1^CON, control treatment, basic diet with no medium chain fatty acids, n = 9. MCFA, MCFA treatment, basic diet and medium chain fatty acids 20 g/d, n = 9.

^2^Pre-calving, from 21 d before calving to calving, Early lactation = from 1 d to 28 d after calving, Peak lactation = from 29 d to 70 d after calving.

^3^BHBA, β-hydroxybutyrate; NEFA, non-esterified fatty acid; TG, triglyceride; INS, insulin.

The interaction between treatment and time-affected lithocholic acid (LCA), hyodeoxycholic acid (HDCA), hyocholic acid (HCA), taurocholic acid (TCA), chenodeoxycholic acid (CDCA), and ursodeoxycholic acid (UDCA) (*P<* 0.01,< 0.01,< 0.01, = 0.03, = 0.03, = 0.06, respectively). Specifically, the addition of MCFAs downregulated the serum concentrations of LCA, HDCA, HCA, and UDCA in MCFA treatment cows 1 d post-calving (*P* = 0.02,< 0.01,< 0.01, = 0.09, respectively, **Table 8**), as well as the serum concentrations of HCA and TCA in cows 14 d post-calving (*P<* 0.01, = 0.01, respectively, **Table 8**). However, CDCA concentration increased significantly 14 d after calving (*P* = 0.02, **Table 8**). LCA also had an upward trend (*P* = 0.08, **Table 8**).

**Table 8:** Effects of feeding medium-chain fatty acid on blood bile acid metabolism of early lactation cows^2^.

Variable^3^ (μ mol/L)	Treatment^1^		SEM	*P*-value		
	CON	MCFA		Treatment	Time	Treatment ×Time
LCA	0.02	0.02	0.002	0.37	<0.01	<0.01
UDCA	0.017	0.015	0.002	0.50	0.04	0.06
HDCA	0.03^a^	0.02^b^	0.002	0.02	0.02	<0.01
CDCA	0.30	0.61	0.118	0.13	0.03	0.03
DCA	1.20	3.47	0.592	0.15	0.05	0.11
HCA	0.05^a^	0.01^b^	0.006	<0.01	<0.01	<0.01
CA	18.77	21.04	3.547	0.71	<0.01	0.27
GLCA	0.08	0.08	0.012	0.81	0.11	0.79
GCDCA	0.82	0.71	0.153	0.70	<0.01	0.33
GDCA	2.03	1.90	0.290	0.80	0.01	0.66
GCA	9.30	6.86	1.328	0.28	<0.01	0.29
TCDCA	0.45	0.43	0.096	0.89	0.10	0.13
TCA	5.02	2.74	0.718	0.08	0.08	0.03
TBA	39.98	38.70	5.626	0.89	<0.01	0.76
CA/CDCA	75.35	89.10	20.696	0.75	0.16	0.85

SEM, standard error of mean.

^a-b^Means within a row with unlike superscripts differ (P ≤ 0.05).

^1^CON, control treatment, basic diet with no medium chain fatty acids, n = 9. MCFA, MCFA treatment, basic diet and medium chain fatty acids 20 g/d, n = 9.

^2^Early lactation, from 1 d to 28 d after calving.

^3^LCA, lithocholic acid; UDCA, ursodeoxycholic acid; HDCA, hyodeoxycholic acid; CDCA, chenodeoxycholic acid; DCA, deoxycholic acid; HCA, hyocholic acid; CA, cholic acid; GLCA, cholic acid; GCDCA, glycochenodeoxycholic acid; GDCA, glycodeoxycholic acid; GCA, glycocholic acid; TCDCA, taurochenodeoxycholic acid; TCA, taurocholic acid; TBA, total bile acid.

### Rumen fermentation

After analysis of rumen fermentation parameters, the concentrations of iso-butyrate and isovalerate in the rumen fluid of dairy cows during the pre-calving period were significantly decreased (*P<* 0.01, 0.01, respectively, **Table 9**), but the total volatile acid content was not affected. Besides, the acetate/propionate had a decreasing trend in early lactation (*P* = 0.08, **Table 9**),

**Table 9:** Effects of feeding medium-chain fatty acid on rumen fermentation of pre-calving, early lactation, and peak lactation cows^2^.

Variable	Treatment^1^		SEM	*P*-value		
	CON	MCFA		Treatment	Time	Treatment ×Time
Pre-calving (mmol/L)						
Acetate	42.34	43.76	1.827	0.71	0.24	0.28
Propionate	8.61	9.93	0.459	0.18	0.49	0.39
Iso-butyrate	0.65^a^	0.52^b^	0.024	<0.01	0.13	0.36
Butyrate	6.12	7.13	0.332	0.16	0.75	0.81
Isovalerate	1.08^a^	0.87^b^	0.035	<0.01	0.05	0.51
Valerate	0.46	0.50	0.022	0.45	0.51	0.50
TVFA^3^	59.25	62.45	2.553	0.55	0.28	0.35
A/P^3^	4.97	4.74	0.074	0.12	0.30	0.25
Early lactation (mmol/L)						
Acetate	45.70	51.17	2.361	0.20	<0.01	0.54
Propionate	16.29	17.64	0.767	0.41	0.73	0.67
Iso-butyrate	0.73	0.60	0.039	0.12	0.77	0.20
Butyrate	7.71	9.15	0.463	0.14	0.21	0.78
Isovalerate	1.34	1.28	0.068	0.69	0.04	0.25
Valerate	0.92	1.09	0.067	0.161	<0.01	0.31
TVFA	75.75	81.47	3.320	0.39	0.05	0.97
A/P	3.14	2.85	0.095	0.08	<0.01	0.14
Peak lactation (mmol/L)						
Acetate	40.09	41.07	1.179	0.67	0.06	0.27
Propionate	15.54	17.18	0.663	0.22	0.09	0.46
Iso-butyrate	0.74	0.75	0.026	0.78	0.33	0.93
Butyrate	6.67	7.07	0.272	0.47	0.20	0.39
Isovalerate	1.45	1.44	0.054	0.87	0.41	0.54
Valerate	0.98	1.12	0.040	0.11	0.87	0.94
TVFA	65.55	69.66	2.090	0.33	0.14	0.42
A/P	2.61	2.52	0.057	0.47	0.23	0.75

SEM, standard error of mean.

^a-b^Means within a row with unlike superscripts differ (P ≤ 0.05).

^1^CON, control treatment, basic diet with no medium chain fatty acids, n = 9. MCFA, MCFA treatment, basic diet and medium chain fatty acids 20 g/d, n = 9.

^2^Pre-calving, from 21 d before calving to calving, Early lactation, from 1 d to 28 d after calving, Peak lactation, from 29 d to 70 d after calving.

^3^TVFA, total volatile fatty acid, A/P, the ratio of acetate to propionate.

The NH_3_-N had an increasing trend in this period (*P* = 0.09). Furthermore, different treatments had significant interaction with NH_3_-N in rumen fluid in terms of time in early lactation (*P* = 0.02), which shows that the concentration of NH_3_-N in MCFA treatment was significantly downregulated 14 d after calving (*P* = 0.04) (**Table 10**). In addition, the pH decreased significantly during early lactation (*P* = 0.01, **Table 10**).

**Table 10:** Effects of feeding medium-chain fatty acid on rumen fermentation and ruminant behavior of pre-calving, early lactation, and peak lactation cows^2^.

Variable	Treatment^1^		SEM	*P*-value		
	CON	MCFA		Treatment	Time	Treatment ×Time
Pre-calving						
NH_3_-N^3^ (mmol/L)	7.03	7.85	0.348	0.09	0.13	0.66
Lactic acid (mmol/L)	3.26	4.34	0.633	0.44	0.99	0.95
pH	6.50	6.50	0.066	0.99	0.15	0.69
Early lactation						
NH_3_-N (mmol/L)	5.58	5.77	0.294	0.69	<0.01	0.02
Lactic acid (mmol/L)	6.26	5.94	0.689	0.81	0.09	0.33
pH	6.54^a^	6.17^b^	0.089	0.01	<0.01	0.67
Rumination (min/d)	522.47	508.25	10.637	0.51	0.08	0.79
Peak lactation						
NH_3_-N (mmol/L)	5.60	4.83	0.243	0.15	0.20	0.34
Rumination (min/d)	528.04	530.51	4.443	0.78	0.48	0.14

SEM, standard error of mean.

^a-b^Means within a row with unlike superscripts differ (P ≤ 0.05).

^1^CON, control treatment, basic diet with no medium chain fatty acids, n = 9. MCFA, MCFA treatment, basic diet and medium chain fatty acids 20 g/d, n = 9.

^2^Pre-calving, from 21 d before calving to calving, Early lactation, from 1 d to 28 d after calving, Peak lactation, from 29 d to 70 d after calving.

^3^NH_3_-N, ammonia nitrogen.

## Discussion

The nutritional metabolism and immune function of dairy cows are interdependent (46). During the transition period, decreased DMI cannot meet cows’ energy needs, leading to the occurrence of NEB and stimulating the mobilization of body fat. Relevant metabolites such as NEFA and BHBA may also affect the function of immune cells, leading to immune dysfunction in dairy cows (47). Also, transition cows go through huge changes in physiological status and various stress factors before and after calving, resulting in the occurrence of excessive inflammatory reactions, which may further affect the metabolic status of animals (48).

In general, the occurrence of inflammatory response is often accompanied by an increase in the concentration of inflammatory cytokines and positive acute phase protein (APP) in the blood. SAA is a signature positive APP in inflammation, and its concentration in the blood is low under healthy conditions, but it increases significantly during the onset of the inflammation (49). Similarly, as an important marker of the inflammatory response, MPO is considered an important tool for diagnosing bacterial infections (50). Compared with CON, the significant decrease of SAA and MPO in dairy cows under MCFA treatment during early lactation may indicate less inflammation in the body. In addition, MCFAs can mediate inflammatory responses by activating GPR84 and TLR-2 (51), promoting proinflammatory cytokine IL-6 production of the immune cell (52), and facilitating the rapid initiation of immune responses to clear pathogens, which may play an important role in alleviating the high stress and infection risk faced by cows during calving (53). Therefore, MCFAs may improve immune cell function and rapidly clear invading pathogens, prevent the occurrence of an excessive inflammatory response, and reduce the incidence of infectious diseases.

The liver function of transition cows often experiences a dramatic change as well. Liver function impairment is considered one of the important reasons for NEB and metabolic disorders in dairy cows. Bile acids are synthesized in the liver and, through interaction with farnesoid X receptor (FXR), regulate digestion, fat metabolism (54), and intestinal inflammation (55). In general, liver reuptake of blood bile acids is reduced, resulting in higher concentrations of bile acids in the blood, which may indicate impaired liver function. Meanwhile, the increasing level of serum bile acid is considered the main characteristic of bile acid siltation. Relevant reports have pointed out that bile acid siltation in animals during pregnancy affects glucose homeostasis. Cholic acid (CA), glycocholic acid (GCA), and taurocholic acid (TCA) are the three bile acids with the highest levels in the blood of dairy cows (56). Our results show that the level of TCA decreased significantly 14 d postpartum and the levels of HDCA, HCA, and LCA decreased significantly at different time points in early lactation. Thus, MCFAs may promote bile acid metabolism and improve the liver function of dairy cows, which plays a vital role in alleviating NEB.

Previous studies have pointed out that MCFAs could improve insulin resistance (57). The increasing level of INS in the prepartum blood of dairy cows may represent reduced sensitivity to insulin receptors in various organs and tissues in the body, thereby reducing insulin sensitivity and triggering insulin resistance (58, 59). The occurrence of insulin resistance reflects that the cows face a certain degree of NEB, which further leads to increased mobilization of body tissues, making cows face greater metabolic pressure and increasing the risk of metabolic diseases. Compared with the cows in the CON group, the concentration of INS in the prepartum blood of cows under MCFA treatment was significantly lower, which may imply that MCFA supplementation can improve the prepartum insulin sensitivity of cows. In addition, the concentrations of BHBA and NEFA in the blood are also numerically down-regulated, and prepartum NEFA and BHBA in the blood are correlated with the occurrence of postpartum metabolic and infectious diseases in dairy cows (60). Furthermore, increased TG concentration in the blood may lead to the deposition of TG in the liver, resulting in the occurrence of fatty liver (61, 62) and adversely affecting the function of the immune system (63). The decreasing trend of the blood TG in our experiment might be linked with the improvement of metabolism. Meanwhile, although the dose of MCFAs applied in this study was small, we cannot exclude the beneficial effect of MCFAs as a fast and efficient energy metabolite on the metabolism regulation of transition cows (64, 65).

The beneficial effects of MCFAs on the immunity and metabolism of dairy cows do not seem to be reflected in productive performance. It has been reported that MCFAs are rapidly absorbed into the liver for metabolism to generate energy and send signals to the brain through the vagus nerve to form satiety signals, thus affecting animal feeding (66). Our results show that MCFA application significantly decreased DMI prepartum, but the DMI in early and peak lactation cows was not affected dramatically. Meanwhile, the prepartum diet consists of higher roughage, which was degraded relatively slowly. So, dairy cows might not adapt to the supplement of MCFAs in the pre-calving period. With regard to the significant decrease in milk production in early lactation, we believe that MCFA supplementation may change the energy allocation of the animal body, and the reduced milk production will be used to reduce the metabolic stress and improve the immune function of cows, thus enhancing the health of cows in the transition period (31). This discrepancy between health and performance needs to be demonstrated in subsequent studies. Furthermore, milk fat percentage increased in both early and peak lactation periods. This is because MCFAs could improve the digestibility of whole digestive tract fat in dairy cows (26).

Although the DMI decreased in the pre-calving period, we observed that the TVFA in rumen fluid saw a numerical increase under MCFA treatment. In other words, MCFA supplementation may improve the rumen function of transition dairy cows, and this effect is more likely based on the beneficial regulation of rumen microbiota and intestinal health. Relevant studies have shown that MCFAs can inhibit intestinal pathogenic microorganisms, enhance intestinal mucosal barrier function, and mediate intestinal immunity (67, 68). IgA plays an important role in defending the enteric pathogenic microorganism passage through the intestinal mucosa, and the decrease in blood Ig A under the MCFA treatment may suggest that the intestinal mucosa is less affected by pathogens (69). The intestinal tract is the largest immune system and plays an important role in resisting the infection of pathogenic microorganisms from feed. MCFAs may improve intestinal health and the microorganism community, which can promote rumen fermentation and prevent systemic inflammation.

## Conclusion

MCFA supplementation can improve the immune function of transition dairy cows to some extent. MCFAs can regulate the level of SAA, MPO, IL-6, TNF-α, and other related immune markers, which will be of benefit in preventing the occurrence of inflammatory diseases. Furthermore, MCFAs might improve the metabolic status of dairy cows in the transition period by modifying liver function and INS sensitivity.

## Data availability statement

The original contributions presented in the study are included in the article/supplementary material. Further inquiries can be directed to the corresponding author.

## Ethics statement

The animal study was reviewed and approved by committee of animal welfare and animal experimental ethical inspection of China Agricultural University. Written informed consent was obtained from the owners for the participation of their animals in this study.

## Author contributions

Conceptualization: WW; methodology: WW and ZC; software: ZW; validation:WW; formal analysis: ZW; investigation: ZW, QW, JY, DL, TX, YZ, ZY, and CG; resources: CL and XS; data curation: ZW; writing (original draft preparation): ZW; writing (review and editing): ZW and WW; visualization: ZW; supervision: WW; project administration: WW; and funding acquisition: WW. All authors have read and agreed to the published version of the manuscript.
